# Establishing energy requirements for body weight maintenance: validation of an intake-balance method

**DOI:** 10.1186/s13104-017-2546-4

**Published:** 2017-06-26

**Authors:** Steven B. Heymsfield, Courtney M. Peterson, Diana M. Thomas, Michael Hirezi, Bo Zhang, Steven Smith, George Bray, Leanne Redman

**Affiliations:** 1 Pennington Biomedical Research Center, LSU System, 6400 Perkins Road, 70808 Baton Rouge, LA USA; 2 University of Alabama, Birmingham, AL USA; 3 United States Military Academy, West Point, NY USA; 4 Translational Research Institute for Metabolism and Diabetes, Florida Hospital, Sanford-Burnham Medical Research Institute, Orlando, FL USA

**Keywords:** Proof study, Metabolic rate, Food intake, Energy expenditure, Doubly-labeled water, Energy balance

## Abstract

**Background:**

Experimentally establishing a group’s body weight maintenance energy requirement is an important component of metabolism research. At present, the reference approach for measuring the metabolizable energy intake (MEI) from foods required for body weight maintenance in non-confined subjects is the doubly-labeled water (DLW)–total energy expenditure (TEE) method. In the current study, we evaluated an energy-intake weight balance method as an alternative to DLW that is more flexible and practical to apply in some settings.

**Methods:**

The hypothesis was tested that MEI from foods observed in a group of subjects maintaining a constant energy intake while keeping their weight within ±1 kg over 10 days is non-significantly different from DLW-measured TEE (TEE_DLW_). Six non-obese subjects evaluated as part of an earlier study completed the inpatient protocol that included a 3-day initial adjustment period.

**Results:**

The group body weight coefficient of variation (X ± SD) during the 10-day balance period was 0.38 ± 0.10% and the slope of the regression line for body weight versus protocol day was non-significant at 1.8 g/day (R^2^, 0.002, p = 0.98). MEI from foods observed during the 10-day balance period (2390 ± 543 kcal/day) was non-significantly different (p = 0.96) from TEE measured by DLW (2373 ± 713 kcal/day); the MEI/TEE_DLW_ ratio was 1.03 ± 0.15 (range 0.87–1.27) and the correlation between MEI from foods and TEE_DLW_ was highly significant (R^2^, 0.88, p = 0.005).

**Conclusions:**

A carefully managed 10-day protocol that includes a constant MEI level from foods with weight stability (±1 kg) will provide a group’s body weight maintenance energy requirement similar to that obtained with DLW. This approach opens the possibility of conducting affordable weight balance studies, shorter in duration than those previously reported, that are needed to answer a wide range of questions in clinical nutrition.

*Trial registration* The study is registered at http://www.clinicaltrials.gov (NCT01672632; August 20, 2012).

**Electronic supplementary material:**

The online version of this article (doi:10.1186/s13104-017-2546-4) contains supplementary material, which is available to authorized users.

## Background

Establishing an experimental group’s maintenance energy requirement is often an important component of metabolic modeling research [1–3]. The current reference approach for objectively measuring the metabolizable energy intake (MEI) from foods required for maintaining a stable body weight is the indirect calorimetry doubly-labeled water (DLW) method that provides a quantitative estimate of total energy expenditure (TEE) [4]. The DLW method involves subjects ingesting a prescribed dose of deuterium and oxygen-18 (^18^O) labeled water and then collecting urine at specified time points over a typical period of 10–14 days. The method is founded on the assumption that TEE and the MEI from foods required for body weight maintenance are equivalent during periods of energy equilibrium or balance. Any changes in energy balance over the DLW period are usually accounted for with measured changes in body weight or body composition [5, 6].

Despite being the reference method for determining TEE in outpatient or non-confined settings, studies employing the DLW method are limited in number. DLW studies typically require clinical research facilities for protocol implementation and specialized laboratory resources for stable isotope analysis that are not widely available. Another limiting factor is that the labeled water isotopes are relatively costly and affordability is a concern for some research groups.

An alternative to the DLW approach for objectively estimating maintenance energy requirements is the “energy intake-weight balance” method [7]. Subjects evaluated with this approach are provided with a diet of precisely known composition, and their intake is titrated up or down until they reach weight “stability”. The implication is that subjects who are weight stable are in near- or at zero energy balance and thus energy intake and expenditure are approximately equal. This state thus characterizes the subject’s energy requirement for weight maintenance. Other than the time involved (days or weeks) and the cost of food purchase and preparation, this approach is potentially accurate and flexible with respect to study facility resources, macronutrient diet composition, the evaluated subject’s metabolic state, and other variables of experimental and clinical interest.

Despite historic and current use, we have not found any reported critical experimental analyses of the energy-intake weight balance method that provided detailed implementation protocols and then tested the validity of the prescribed approach. Energy and elemental balance methods were used in experimental protocols more than one century ago by Atwater [8, 9] and later by Reifenstein [10] and others several decades ago [11–14]. Of those recently using the method, an example is provided by the studies of Rosenbaum et al. [7] in which subjects are “titrated” to weight stability and then held at a fixed MEI from foods until the slope of body weight versus study day approaches zero, empirically defined as equal to ±10 g/day or less for 14 days. When combined with an adjustment period, the duration of these body weight maintenance experiments conducted on a clinical research unit often extends for 4–6 weeks [7].

In the current report, for the first time, we evaluated the validity and clinical features of an intake-balance approach to estimating body weight maintenance energy requirements by examining the energetics of a 10-day protocol in which subjects were maintained on a constant energy intake and whose body weight varied by less than ±1 kg. Our findings suggest that for small subject groups this relatively simple approach provides energy intake estimates non-significantly different from those measured by DLW.

## Methods

### Subjects and experimental design

The current inpatient study includes a rigorously evaluated subgroup of subjects previously reported by Bray et al. [15] that examined topics unrelated to the present report. The specific hypothesis tested was that subjects maintaining a constant MEI from foods and stable body weight (±1 kg) over 10 days are in energy balance and thus the observed MEI is equivalent to TEE as measured by DLW.

Study entry criteria included a body mass index (BMI) of 19–30 kg/m^2^, an age of 18–35 years, and the absence of recent weight change or chronic disease. The protocol was approved by the Pennington Biomedical Research Center (PBRC) Institutional Review Board, conducted in accord with the Declaration of Helsinki, and all subjects signed an informed consent prior to participation. The study is registered at http://www.clinicaltrials.gov (NCT01672632).

The Proof Study included a 3-day adjustment period that provided a transition for subjects moving from their usual diets to the inpatient diet and activity level [15]. The subjects then entered the weight stabilization phase and those who maintained their weight within ±1 kg during this 5-day period were then entered into a 5-day energy balance period during which their weight was required to remain stable at ±1 kg while energy intake was held constant. The study hypothesis was tested in six subjects enrolled in the current study who maintained both a stable body weight ±1 kg and a constant energy intake over the 10-day combined weight stabilization and energy balance periods concurrent with a DLW evaluation of TEE.

The six subjects included in the current study thus all were evaluated over 13 consecutive days. The first 3 days were an adjustment period on the PBRC Metabolic Unit and during the last 10 days they maintained a stable body weight (±1 kg) and constant energy intake with concurrent TEE evaluation by DLW.

### Study protocol

Participants completed a dual-energy X-ray absorptiometry (DXA; Hologic QDR with Windows software version V11.1; Hologic Inc., Bedford, MA) study for estimation of total body fat mass (FM) and fat-free mass (FFM) prior to admission. Participants were then admitted to the PRBC Metabolic Unit for the 3-day adjustment period during which they were fed an approximate weight maintenance diet, which was estimated as 1.4× resting energy expenditure (REE) calculated using an equation incorporating DXA-derived FM and FFM (Additional file 1). On the third day of the adjustment period, participants spent 23 h inside the PBRC Metabolic Chamber, during which time total energy expenditure (TEE_CH_) was measured. The TEE_CH_ measurement was extrapolated to 24 h with results expressed in kcal/day. Subjects were fed 85% of their metabolic unit energy expenditure requirement on the chamber day to compensate for reduced activity levels.

After discharge from the metabolic chamber, participants followed the 5-day weight stabilization protocol with energy intake set at 1.15 × TEE_CH_. The 15% increase in prescribed energy intake above that estimated in the chamber was intended to compensate for the greater physical activity related to the more sedentary activity observed during the metabolic chamber test. Subjects evaluated in the current report then maintained that level of energy intake for the next 10 days while body weight varied within ±1 kg. Subjects during this period remained relatively inactive on the metabolic unit without any added exercise programs.

### Diet protocol

Subjects were fed a standard diet consisting of 15% protein, 25% fat, and 60% of energy from carbohydrates as three meals and snacks throughout the 13 protocol days. Meals were prepared in duplicate, and the duplicate meal was analyzed by Covance Laboratories (Princeton, NJ) for fat, protein, and carbohydrate content. Specific foods provided during the weight stabilization and energy balance phases were rotated on a 5 day basis. Metabolizable energy intake values were calculated using 4 kcal/g for protein, 9 kcal/g for fat, and 4 kcal/g for carbohydrate. The dietary staff supervised meals to confirm that all foods were eaten; no additional foods, salt, or caffeine intake were allowed. A multivitamin was taken daily by each subject throughout the study.

Body weight was measured post-void upon arising before breakfast each day with subjects clothed in a paper gown. Metabolic or nude weight was calculated by subtracting the gown weight from the total measured weight.

### Energy expenditure

#### Metabolic chamber

Total energy expenditure was measured in the PBRC Metabolic Chamber as previously described [16]. Oxygen and carbon dioxide levels in the chamber were measured using, respectively, a Magnos 4G magneto-pneumatic oxygen analyzer and a Uras 3G infrared CO_2_ analyzer (Hartmann and Braun, Frankfurt am Main, Germany) with gas sampling rates of 60 Hz. Energy expenditure (TEE_CH_) was calculated from VO_2_, VCO_2_, and 24-h urinary nitrogen excretion using the Weir equation [17].

#### Doubly-labeled water

Day 1 of the DLW period coincided with the first weight stabilization day and the last day coincided with completion of the energy balance period. Details of the DLW protocol are presented in Bray et al. [15]. Carbon dioxide production was calculated from the DLW data as

1 $$ {\text{rCO}}_{ 2} = \left( {{\text{N}}/ 2.0 7 8} \right)\left( { 1.00 7 {\text{ k}}_{\text{O}} - 1.0 4 1 {\text{ k}}_{\text{H}} } \right) - 0.0 2 4 6 {\text{ r}}_{\text{GF}} $$

with rCO_2_ carbon dioxide production rate (moles/day), N total body water calculated as N_O_/1.007, k_O_ and K_H_ oxygen-18 and deuterium the elimination rates from total body water, and R_GF_ the fractionated gaseous water loss rate calculated as 1.05 N (1.007 k_O_ − 1.041 k_H_). Total energy expenditure in kcal/day was then calculated as

2 $$ {\text{TEE}}_{\text{DLW}} = 2 2. 4 {\text{ rCO}}_{ 2} ( 3. 9/{\text{respiratory quotient}} + 1. 10) $$

#### Predicted energy expenditure

Those using the energy-intake weight balance method in the future may not have access to the extensive energy expenditure evaluation technologies used in the Proof Study [15]. Accordingly, to expand the energy-intake weight balance method for use in studies at sites that do not have these resources, we examined in the current study the associations between the MEI from foods required for weight maintenance established during the 10-day balance period and three commonly used representative REE prediction equations (Harris–Benedict [HB], Livingston–Kohlstadt [LK], Mifflin–St. Jeor [MS]) [18–21] and the National Academy of Sciences (NAS) TEE prediction equation https://en.wikipedia.org/wiki/Institute_of_Medicine_Equation). The details of these prediction equations, all based on body weight, height, age, sex, and physical activity level for TEE are described in detail in Additional file 1.

### Statistical methods

The coefficient of variation (CV; %) and standard deviation (SD) for the observed body weights were derived over the 10-day evaluation period for each subject and all subjects combined. Similar to Rosenbaum et al. [7], we derived the slope of body weight versus day for each subject over the ten evaluation days and for all subjects combined using linear regression analysis. The study hypothesis was tested by comparing the mean ± SD MEI from foods observed over the 10-day evaluation period to the corresponding TEE_DLW_ using a paired *t* test with significance set at p < 0.05. We also derived the ratios (mean ± SD) of MEI to TEE_DLW_, predicted REEs, and predicted TEE_NAS_ values. The correlations between these different measures of energy exchange were also evaluated using linear regression analysis. Results are expressed as the mean SD.

## Results

### Subjects

The characteristics of the six evaluated subjects are presented in Table 1. There were 3 males and 3 females who ranged in age from 25 to 35 years and in BMI from 23.1 to 27.9 kg/m^2^.

**Table 1:** Subject characteristics

Subject (sex/race)	Age (years)	Weight (kg)	Height (cm)	BMI (kg/m^2^)	%fat
A (F/Bl)	29	66.8	158.9	26.5	35.4
B (F/W)	22	64.3	166.3	23.2	28.0
C (F/Bl)	25	53.7	151.7	23.4	24.8
D (M/Bl)	25	76.1	181.5	23.1	17.7
E (M/W)	25	90.2	178.2	28.4	29.6
F (M/Bl)	35	106.2	195	27.9	20.2
Mean ± SD	26.8 ± 4.6	76.2 ± 19.2	171.9 ± 16.0	25.4 ± 2.5	25.9 ± 6.5

*Bl* black, *BMI* body mass index, *F* female, *M* male, *SD* standard deviation, *W* white

### Body Weight

The mean group body weight CV and SD during the 10-day balance period were 0.38 ± 0.10% (range 0.25–0.53%) and 0.29 ± 0.10 kg (range 0.16–0.42 kg), respectively (Table 2). The correlation between the 5-day weight stabilization and 5-day energy balance period CVs (R^2^, 0.019) was non-significant (p = 0.50).

**Table 2:** Body weight evaluations during the 10-day protocol

Subject	Body weight^a^		Body weight versus day^b^	
	CV (%)	SD (kg)	Slope	Intercept (R^2^)
A	0.25	0.16	−20.0	66.2 (0.14)
B	0.53	0.36	6.2	63.4 (0.32)*
C	0.31	0.16	21.8	52.6 (0.16)
D	0.44	0.33	41.2	76.2 (0.14)
E	0.34	0.31	1.8	89.7 (0.001)
F	0.39	0.42	−103.0	108.1 (0.55)**
Mean ± SD	0.38 ± 0.10	0.29 ± 0.10	1.0	76.0 (0.0002)

* p < 0.10 and ^**^ = 0.01

^a^CV, coefficient of variation and standard deviation (SD) in body weight over the 10 day protocol

^b^Slope (g/day), intercept (kg), and R^2^ for regression of body weight on protocol day

The slopes of the regression of body weight on protocol day varied from −103 g/day (p = 0.09) in Subject F to 41.2 g/day (p = 0.46) in Subject D. The group as a whole had a regression line slope of 1.8 g/day with an R^2^ of 0.002, p = 0.98.

**Fig. 1:**
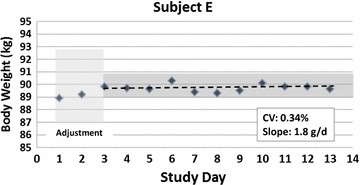
Body weight versus protocol day in representative Subject E. The body weight coefficient of variation (CV) during the 10-day protocol was 0.34% and the slope of body weight versus day regression line was 1.8 g/day

**Fig. 2:**
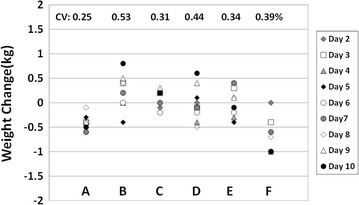
Difference in body weight from day 1 of the weight stabilization period and each of the 9 remaining protocol days in subjects A through F. The corresponding body weight coefficient of variation (CV, %) is shown in the *figure*

An example of the observed body weight over the 10-day study period is shown in Fig. 1 for Subject E. This subject’s body weight was 89.7 kg on the first balance day with maximum and minimum weights over the remaining 9 days of 90.3 and 89.6 kg, respectively (CV = 0.34%; SD = 0.31 kg; regression line slope, 1.8 g/day; R^2^, 0.003, p = 0.99). The variability in body weight from day 1 of the weight stabilization period is shown for all six subjects in Fig. 2.

### Energy expenditure

The results of MEI and energy expenditure evaluations are shown in Table 3. The table includes the ratio of MEI during the balance period to respective TEE_DLW_ and the calculated REE and TEE_NAS_ estimates that could be employed for studies at centers without access to indirect calorimeters.

**Table 3:** Metabolizable energy intake and energy expenditure results

Subject	MEI	TEE_DLW_^a^	TEE_NAS_	REE_HB_	REE_LK_	REE_MS_
A	2007	1582 (1.27)	1967 (1.02)	1427 (1.41)	1394 (1.44)	1350 (1.49)
B	2007	2299 (0.87)	2026 (0.99)	1455 (1.38)	1401 (1.43)	1403 (1.43)
C	1906	1642 (1.16)	1793 (1.06)	1296 (1.47)	1266 (1.51)	1188 (1.60)
D	2407	2410 (1.00)	2615 (0.92)	1838 (1.31)	1765 (1.36)	1766 (1.36)
E	2704	2871 (0.94)	2799 (0.97)	2005 (1.35)	1906 (1.42)	1882 (1.44)
F	3310	3434 (0.96)	3013 (1.10)	2266 (1.46)	2013 (1.64)	2114 (1.57)
R^2^ value^b^	NA	0.88	0.90	0.96	0.88	0.92
Mean ± SD	2390 ± 543	2373 ± 713 (1.03 ± 0.15)	2369 ± 504 (1.01 ± 0.06)	1714 ± 382 1.40 (0.06)	1624 ± 310 (1.47 ± 0.10)	1617 ± 358 (1.48 ± 0.09)

*REE* resting energy expenditure estimated by Harris–Benedict (HB; [18]), Livingston–Kohlstadt (LK; [20]), and Mifflin–St. Jeor (MS; [21]) equations; *MEI* metabolizable energy intake; *NA* not applicable; *TEE* total energy expenditure by doubly-labeled water (DLW) and National Academy of Science (NAS) prediction equations (https://en.wikipedia.org/wiki/Institute_of_Medicine_Equation)

^a^In brackets, ratio of MEI to measured or predicted energy expenditure. All energy term units are in kcal/day

^b^R^2^ value for MEI versus measured or predicted energy expenditure. All, p < 001

**Fig. 3:**
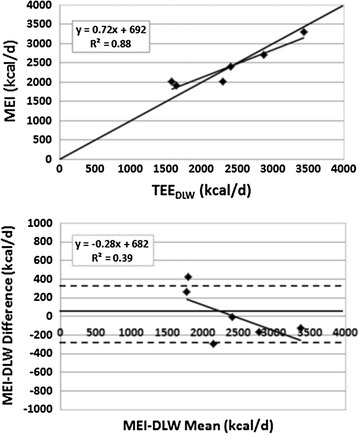
*Upper panel* metabolizable energy intake (MEI) from foods versus total energy expenditure by doubly-labeled water (TEE_DLW_) observed over the 10 days study balance period. The correlation between the two (R^2^, 0.88) was significant at p = 0.005. *Lower panel* Bland–Altman plot of between method MEI-DLW differences versus mean of the two methods (R^2^, 0.39, p = 0.19). ±2SD from the method mean (*solid horizontal line*) are shown as *dashed lines* in the figure

There was no significant difference (p = 0.96) between MEI from foods observed during the 10-day balance period (2390 ± 543 kcal/day) and TEE measured by DLW (2373 ± 713 kcal/day) and there was a high significant correlation between the two energy expenditure measures (R^2^, 0.88, p = 0.005) (Fig. 3, upper panel). A Bland–Altman plot of the between-method difference (MEI from foods-TEE_DLW_) was non-significant (R^2^, 0.39; p = 0.19) (Fig. 3, lower panel). The ratio of MEI to TEE_DLW_ was 1.03 ± 0.15 (range 0.87–1.27) and the difference between MEI and TEE_DLW_ was non-significantly (p > 0.05) correlated with the regression line slope of body weight versus day over the 10-day balance period.

The results for the evaluated REE and TEE prediction equations are presented in Table 3. Metabolizable energy intake was highly correlated with the three predicted values for REE (R^2^s, 0.96, 0.88, and 0.92 for HB, LK, and MS; all p < 0.01). The ratio of MEI to predicted REE values were, respectively, 1.40 ± 0.06, 1.47 ± 0.10, and 1.48 ± 0.09 for HB, LK, and MS equations. The corresponding R^2^ for the correlation between MEI and TEE_NAS_ estimates was 0.90 (p = 0.004) with a MEI/TEE_NAS_ ratio of 1.01 ± 0.06.

## Discussion

One of the oldest questions in the study of human energy metabolism is how to precisely estimate a subject or group’s maintenance energy requirements. The maintenance energy requirement can be defined as the MEI level from foods needed to balance TEE and thus maintain body energy stores and balance stable over time. Here we show that a relatively straight forward and practical energy-intake weight balance protocol provides almost identical group mean MEI values to those estimated from energy expenditure measured using the DLW method for a relatively small sample of six subjects.

While long recognized as a viable means of estimating maintenance energy requirements, the energy-intake weight balance method has had little use in current years with the exception of the extensive carefully controlled balance studies reported by Rosenbaum et al. [7]. In these experiments the author’s employed the method for long adjustment and measurement periods (4–6 weeks) with stringent body weight stability criteria (weight change <10 g/day over 14 days) to critically evaluate energy balance regulatory mechanisms in adults confined to a clinical research unit [1, 22]. Our findings indicate that group mean MEI values from foods for body weight maintenance comparable to those of the DLW method can be obtained over a 10-day period during which energy intake is maintained constant and body weight remains within ±1 kg of that on day 1.

The six subjects included in the current report completed the full evaluation over 13 days: 3 days for adjustment and 10 days for the balance experiment. An additional 17 subjects enrolled in the proof study [15] maintained a stable body weight (±1 kg) during the weight stabilization period, although small adjustments (±200 kcal/day) were empirically made in their energy intake and as a group they gained weight over the full 10-day protocol with DLW. However, all of these subjects had stable energy intakes and body weights during the 5-day energy balance period. Adding on another 5 days of energy intake and weight stability to the protocol in these subjects would likely thus have extended their full weight maintenance evaluation to 18 days [15]. These observations suggest that comparable results to those in the current report can be achieved over 2- to 3-week periods in most subjects.

While our findings do not fully negate the complexity and attention to detail needed for completion of metabolic studies, the energy-intake weight balance method offers experimental opportunities that are importantly more practical and can be implemented more widely than the DLW approach. The energy-intake balance method can be conducted in outpatient settings, a wide array of options are available for provided foods (i.e., liquid, solid; variable macronutrient proportions; commercial offerings; etc.), protocols can vary in level of inactivity or prescribed exercise, and people who are healthy or chronically ill can be studied. Our studies were conducted in a highly controlled environment and it is likely that real-world evaluations may be more difficult to manage. Evaluation of obese, medically-compromised, or actively exercising subjects outside of a metabolic unit may prove more complex. On the other hand, our study was not originally designed to specifically evaluate the energy-intake weight balance method. The possibility exists for non-metabolic unit evaluated subjects to measure body weight and keep food/activity records for long time periods before formally starting the balance experiment. Three critical elements to successfully conduct this kind of study are needed: compliant and/or monitored subjects, liquid or solid foods of known macronutrient and energy content, and accurate body weight scales.

With further development and testing, the energy-intake weight balance approach might also be valid at the individual level as would be of interest in the growing personalized health and self-monitoring movements [23]. Assuming the subject would be compliant out of self-interest, known composition prepared solid or liquid meals are easily acquired at stores in most settings. Accurate body weight scales, often with Bluetooth and internet capabilities that can facilitate recording and tracking, are widely available and relatively inexpensive. Another potential variation in the method would be if an indirect calorimeter for measuring REE is available that could facilitate initial estimates of MEI values for weight maintenance. A measured value for REE would also allow calculation of physical activity level values (MEI established over the 10-days/measured REE) upon completion of the study. Small, relatively inexpensive indirect calorimeters that in some cases couple with cellular telephones are becoming available.

A key aim of our study was to see if the empirically derived protocol allowed us to estimate MEI values from foods for body weight maintenance similar to those from the DLW method. In fact, early validation studies of the DLW method were based on variations of the intake-balance method that included metabolic chamber and body composition evaluations in relatively small samples such as in the present report [24, 25]. The correlation between MEIs derived in our study by the intake-balance method and that by DLW (R^2^, 0.88) is similar in magnitude to those reported in these earlier studies [25, 26].

One long-held concern when comparing these approaches is the possibility that small gains or losses in body energy stores may occur that cannot be accurately detected with currently available body composition methods. Our group mean change in body weight over the 10 day protocol impressively approached zero (~2 g/day) as is required for individual subjects in the rigorous method of Rosenbaum et al. (i.e., <10 g/day) [22]. Our group body weight CV over the 10-day protocol was relatively small (0.38 ± 0.10%) and is very similar in magnitude to that reported in a series of publications on this topic reported by Edholm [27], Khosla and Billewicz [28], and Robinson and Watson [29] that established body weight and energy balance variation under stable conditions in healthy adults. Through careful observation and experimentation these authors found that body weight can vary up to 1 kg under stable living conditions, that energy balance and long-term weight changes are significantly correlated, and that day-to-day changes in body weight are largely due to changes in water balance. Garrow [30] concluded from these studies that at best energy balance can be established within ±50 kcal/day. This inherent variability in body weight, water, sodium [31], and energy balance [13] poses a challenge to establishing optimum protocol design and sample size for the energy-intake balance method and our approach should only be considered a first step in developing a practical experimental design.

As part of our study we evaluated the relations between MEI from the intake-balance method and estimated REE and TEE values from some representative energy expenditure prediction equations. With respect to REE, we found that actual MEI from foods observed during the 10-day protocol was on average 1.40–1.48 times calculated REE (individual subject range across all equations, 1.31–1.64) depending on the selected prediction model. These mean values and range are very similar to the energy requirements for body weight maintenance typically used in metabolic unit studies [12]. Similarly, we found a good correlation (R^2^, 0.90) and a close mean value between MEI from foods and TEE from the NAS estimated energy requirement prediction equations (ratio 1.01 ± 0.06). Starting levels for energy intake on metabolic units can thus be derived using these predictions while free-living TEE estimates can be calculated using the NAS equations summarized in the Additional file 1. While these predictions may be of use in setting initial energy requirements for weight maintenance, an alternative is to acquire this information in highly compliant subjects from food and activity records maintained prior to study.

## Conclusions

In sum, we show that a carefully managed 10-day protocol with subjects maintaining a constant MEI and weight stability (±1 kg) can provide a group’s energy requirement for body weight maintenance comparable to that estimated from the DLW method. Our approach opens the possibility of conducting practical weight balance studies, shorter than those previously reported, that are needed to answer a wide range of questions in clinical nutrition.

## Additional file

**Additional file 1.** Additional information.

## Abbreviations

BMI: body mass index
CV: coefficient of variation
DLW: doubly-labeled water
FFM: fat-free mass
FM: fat mass
NAS: National Academy of Sciences
REE: resting energy expenditure
SD: standard deviation
TEE: total energy expenditure
