# Supplementary material for: Establishing energy requirements for body weight maintenance: validation of an intake-balance method
Source: BMC Res Notes. 2017 Jun 26;10:220. doi: 10.1186/s13104-017-2546-4 (PMC5485536; doi:10.1186/s13104-017-2546-4)
Supplement: Supplementary file 1 — Additional file 1. Additional information. [file 13104_2017_2546_MOESM1_ESM.docx]

**ADDITIONAL INFORMATION**

The energy intake during the adjustment phase was calculated by averaging two resting energy expenditure (REE, kcal/d) prediction equations [15] and then multiplying by a physical activity level factor between 1.3-1.4:

REE = [538 + 25 (FFM) + 5.5 (FM)], (A.1)
and
 REE = 1294 - 7 (age) + 33 (FFM) + 3 (FM) for women, (A.2A)
and

REE = 1294 - 7 (age) + 33 (FFM) + 3 (FM) + 92 for men. (A.2B)

A 10% reduction in energy intake estimates for men and 12% for women was used to compensate for limited activity on the Metabolic Ward. These equations and approach were chosen based on previous experience with metabolic ward studies. Body composition was measured with DXA and units are in kg.

Three REE prediction equations were evaluated in the current study along with one TEE prediction equation as shown in the table below. In the current study we assumed a “sedentary” physical activity level when deriving energy requirements on the Metabolic Unit.

| Equation (ref) | Men | Women |
| --- | --- | --- |
| Harris-Benedict [18] | REE=66.5+(13.75xBW)+ (5.003xHt)-(6.775xA) | REE=655.1+(9.563xBW)+ (1.850xHt)-(4.676xA) |
| Livingston-Kohlstadt [20] | REE=293xBW^0.4330^- (5.92xA) | REE=248xBW^0.4356^- (5.09xA) |
| Mifflin-St. Jeor [21] | REE=10xBW+6.25xHt-5xA+5-161 | REE=10xBW+6.25xHt-5xA |
| National Academy of Sciences^† *^ | EER=662-(9.53xA)+PAx(15.91xBW+539.6xHt) | EER=354-(6.91xA)+PAx(9.36xBW+726xHt)) |

Abbreviations: A, age, in years; BW, body weight in kg; EER, estimated energy requirement in kcal/d; Ht, height in cm or m^†^; PA, physical activity level [see table below]; REE, resting energy expenditure in kcal/d. *<https://en.wikipedia.org/wiki/Institute_of_Medicine_Equation>.

| Physical Activity Level | Men | Women | Comment |
| --- | --- | --- | --- |
| Sedentary | 1 | 1 | Light physical activity associated with independent living. |
| Moderately Active | 1.11 | 1.12 | About half an hour of moderate to vigorous exercise in addition to sedentary activity. |
| Active | 1.25 | 1.27 | At least an hour of exercise. |
| Very Active | 1.48 | 1.45 | Being physically active for several hours each day. |
